# Genetic loci and key candidate genes associated with fatty acid composition in the breast muscle of Sansui ducks identified through GWAS

**DOI:** 10.1186/s12864-026-12905-6

**Published:** 2026-05-07

**Authors:** Yulong Feng, Mengru Xu, Meijuan Li, Yuxi Lu, Guotao Dai, Chengcheng Tian, Yu Zhao, Hehe Liu, Anqi Huang

**Affiliations:** 1 Institute of Animal Husbandry and Veterinary Medicine, Guizhou Academy of Agricultural Sciences, Guiyang, Guizhou Province China; 2 Guizhou Provincial Key Laboratory of Livestock and Poultry Genetic Resources Innovation and Utilization, Guiyang, Guizhou China; 3 Farm Animal Genetic Resources Exploration and Innovation Key Laboratory of Sichuan Province, Sichuan Agricultural University, Chengdu, Sichuan 611130 China

**Keywords:** Sansui duck, Fatty acid composition, Breast muscle, GWAS, Candidate gene

## Abstract

**Background:**

The composition of fatty acids, particularly the content of unsaturated fatty acids (UFA), is closely related to meat quality and flavor in ducks.

**Result:**

In this study, 38 fatty acids were detected in the breast muscle of Sansui ducks, yielding 50 distinct fatty acid composition traits. The results showed that the predominant fatty acids were C18:1n-9, C16:0, C18:2n-6, C18:0, and C20:4n-6. Moreover, the breast muscle of Sansui duck was rich in UFA, accounting for 64.01% of the total fatty acids. Genome-wide association analysis (GWAS) identified 158 single nucleotide polymorphisms (SNPs) significantly associated with fatty acid composition traits, which were annotated to 70 protein-coding genes, including *LDLRAD3*, *ACVR1*, *FGF6*, and *CASQ2*. Additionally, linkage disequilibrium (LD) analysis revealed a candidate region (10,533,318 − 10,546,189 bp) on chromosome 14, where *NSG2* was identified as a key candidate gene influencing C17:1 content. Functional enrichment analysis suggested that pathways such as Adherens junctions and MAPK signaling may play significant roles in fatty acid biosynthesis.

**Conclusion:**

These findings reveal the genetic structure of fatty acids composition in the breast muscle of Sansui duck, providing strong evidence for understanding the genetic regulatory mechanisms of fatty acid composition.

**Supplementary Information:**

The online version contains supplementary material available at 10.1186/s12864-026-12905-6.

## Background

Poultry, along with pork and beef, serves as a major source of dietary protein. With societal development and improved living standards, poultry production has irreversibly shifted from quantity growth to quality enhancement [1]. The composition and content of fatty acids are important factors that influence meat quality and flavor. Among these, the content of unsaturated fatty acids (UFAs) is a key factor affecting meat quality and flavor, as they can be converted into various derivatives within the body, altering cellular signaling, anti-inflammatory responses, and other biological processes [2]. Moreover, increasing the dietary intake of UFAs while reducing the intake of saturated fatty acids (SFAs) can significantly improve blood lipid profiles and lower the risk of cardiovascular diseases and type II diabetes [3].Duck breast is rich in UFAs, which are essential nutrients that humans cannot synthesize endogenously [4].Therefore, it is of great significance to study the composition and content of fatty acids in duck pectoral muscle and reveal their genetic mechanisms to improve the nutritional value and flavor of duck meat.

The composition of fatty acids is a complex quantitative trait, and the application of modern omics technologies has provided strong support for the genetic analysis of this trait. In livestock, the candidate genes and regulatory loci for muscle fatty acid composition traits have been extensively studied [5–7]. These studies indicate that the composition of muscle fatty acids is co-regulated by multiple genes, and regulatory loci located on different chromosomes jointly regulate multiple fatty acid traits. In poultry, several candidate genes affecting the proportion of PUFA in the breast muscles of Huangshan Black Chicken have been identified through RNA-seq screening, such as *OGN*, *FADS2*, *CYTL1*, and *ADGRD*1 [8]. Weighted gene co-expression network analysis (WGCNA) revealed two differentially expressed circular RNAs and two competitive endogenous RNAs that could regulate the differentiation of fat formation in chickens [9, 10]. Fan used genome-wide association analysis (GWAS) and WGCNA strategies to study the fatty acid composition traits in the breast muscles of Gushi chickens and identified two significant single nucleotide polymorphisms (SNPs) that explain 5.6% of the phenotypic variation of PUFA, as well as nine important potential candidate genes [11]. Jin detected 30 quantitative trait loci (QTLs) related to fatty acid composition traits in chickens [12], though only seven QTLs related to fatty acids are currently recorded in the chicken QTL database. These studies have deepened our understanding of the genetic regulation of fatty acid composition traits in poultry, but genetic studies on fatty acid composition in duck muscle are still limited.

Sansui duck is a renowned local breed from Guizhou, celebrated for its tender, flavorful meat, high amino acid content, and low muscle cholesterol levels. To explore the superior traits of Sansui duck breast muscle, we measured the content of 38 types of fatty acids in the breast muscle of this population and used them as phenotypes for GWAS analysis. This approach enabled us to uncover the genetic structure and key regulatory genes associated with the fatty acid composition of the breast muscle. This study provides valuable insights into the genetic regulation of fatty acid composition in Sansui duck breast muscle from the perspective of genetic variation and offers a reference for the molecular mechanisms underlying meat quality traits.

## Results

### Fatty acids composition of breast muscle in Sansui duck

Based on the contents of 38 fatty acids in the breast muscle of the Sansui ducks, 12 fatty acid traits were calculated, resulting in a total of 50 phenotypic traits related to fatty acid composition (Table 1). Among these, oleic acid (C18:1n-9) exhibited the highest content, followed by palmitic acid (C16:0), linoleic acid (C18:2n-6), and stearic acid (C18:0), together accounting for approximately 82.35% of the total fatty acid content. The breast muscle of Sansui duck is rich in UFA (64.01%), 1.79 times that of SFA. The total content of PUFA was lower than that of MUFA, with a PUFA/MUFA ratio of 0.77. Additionally, n-6 and n-3 fatty acids accounted for 24.73% and 1.18% of the TFA, respectively, with an n-6/n-3 ratio of 21.13.

**Table 1:** Summary statistics for fatty acid compsition traits in in breast muscle of the Sansui duck

Category	Trait	Mean ± SD (µg/g)	Proportion (%)
SFA	C6:0	1.36 ± 0.65	0.04
	C8:0	0.35 ± 0.13	0.01
	C10:0	0.44 ± 0.11	0.01
	C11:0	0.35 ± 0.12	0.01
	C12:0	1.31 ± 0.33	0.03
	C13:0	0.30 ± 0.05	0.01
	C14:0	12.77 ± 5.12	0.30
	C15:0	1.45 ± 0.38	0.04
	C16:0	863.66 ± 266.74	20.59
	C17:0	3.38 ± 0.75	0.08
	C18:0	601.50 ± 134.06	14.69
	C20:0	3.67 ± 1.08	0.09
	C21:0	0.57 ± 0.11	0.01
	C22:0	1.61 ± 0.53	0.04
	C23:0	0.75 ± 0.16	0.02
	C24:0	0.86 ± 0.27	0.02
MUFA	C14:1	1.23 ± 0.42	0.03
	C15:1	0.60 ± 0.11	0.02
	C16:1	78.56 ± 37.19	1.82
	C17:1	12.92 ± 3.47	0.32
	C18:1n-9	1361.43 ± 542.54	31.84
	C18:1n-7	96.74 ± 49.64	2.29
	C20:1	9.46 ± 3.42	0.23
	C22:1n-9	1.76 ± 0.44	0.04
	C24:1	0.93 ± 0.30	0.02
PUFA	C18:2n-6	638.09 ± 202.87	15.24
	C18:3n-6	1.57 ± 0.41	0.04
	C18:3n-3	9.32 ± 3.53	0.22
	C20:2	7.39 ± 1.76	0.18
	C20:3n-3	0.68 ± 0.13	0.02
	C20:3n-6	10.70 ± 2.86	0.26
	C20:4n-6	331.91 ± 85.94	8.16
	C20:5n-3	0.96 ± 0.24	0.02
	C22:2	0.74 ± 0.24	0.02
	C22:4	52.17 ± 13.30	1.29
	C22:5n-3	11.51 ± 2.99	0.28
	C22:5n-6	42.08 ± 16.78	1.03
	C22:6n-3	25.96 ± 10.13	0.63
Statistical	TFA	4191.03 ± 1258.24	100.00
	SFA	1494.32 ± 387.20	35.99
	UFA	2696.71 ± 879.12	64.01
	MUFA	1563.63 ± 617.51	36.61
	PUFA	1133.08 ± 306.71	27.40
	UFA/SFA	1.79 ± 0.14	/
	PUFA/SFA	0.76 ± 0.09	/
	MUFA/SFA	1.02 ± 0.15	/
	PUFA/MUFA	0.77 ± 0.18	/
	n-3	48.43 ± 14.29	1.18
	n-6	1024.35 ± 283.31	24.73
	n-6/n-3	21.13 ± 3.51	/

*Abbreviations*: *SFA* Saturated fatty acids, *MUFA* Monounsaturated fatty acids, *PUFA* Polyunsaturated fatty acids, *UFA* Unsaturated fatty acids, *TFA* Total fatty acid content. Proportion refers to the ratio of each fatty acid content to the total fatty acid content

**Fig. 1:**
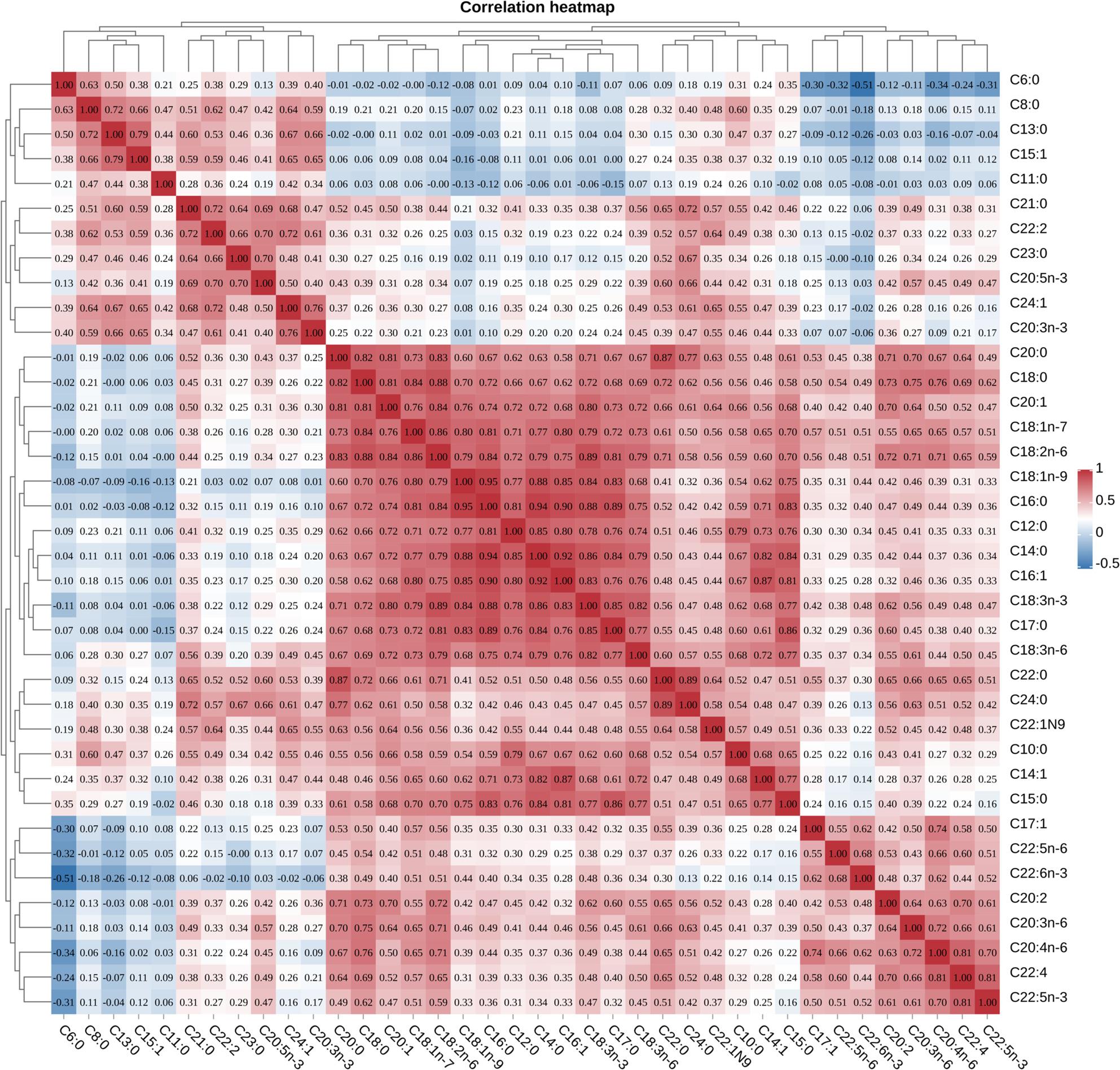
Correlation analysis of 38 fatty acid contents. The colors (numbers) represent the pairwise correlation coefficients of the fatty acid content traits. Red indicates a positive correlation, and blue indicates a negative correlation

Phenotypic correlations among the 38 fatty acid content traits are presented in Fig. 1. The saturated fatty acid traits showed relatively high correlations with each other, and fatty acids with the same number of carbon atoms also exhibited stronger correlations. Notably, strong correlations (*r* > 0.5) were observed among 13 fatty acid traits, including C12:0, C14:0, C16:0, C16:1, C17:0, C18:0, C18:1n-9, C18:1n-7, C18:2n-6, C18:3n-3, C18:3n-6, C20:0, and C20:1, which is consistent with the biochemical principles of fatty acid interconversion. Furthermore, the most abundant fatty acid, C18:1n-9, showed strong positive correlations with C16:0, C14:0, and C16:1.

### Comparison of fatty acid content between males and females

**Fig. 2:**
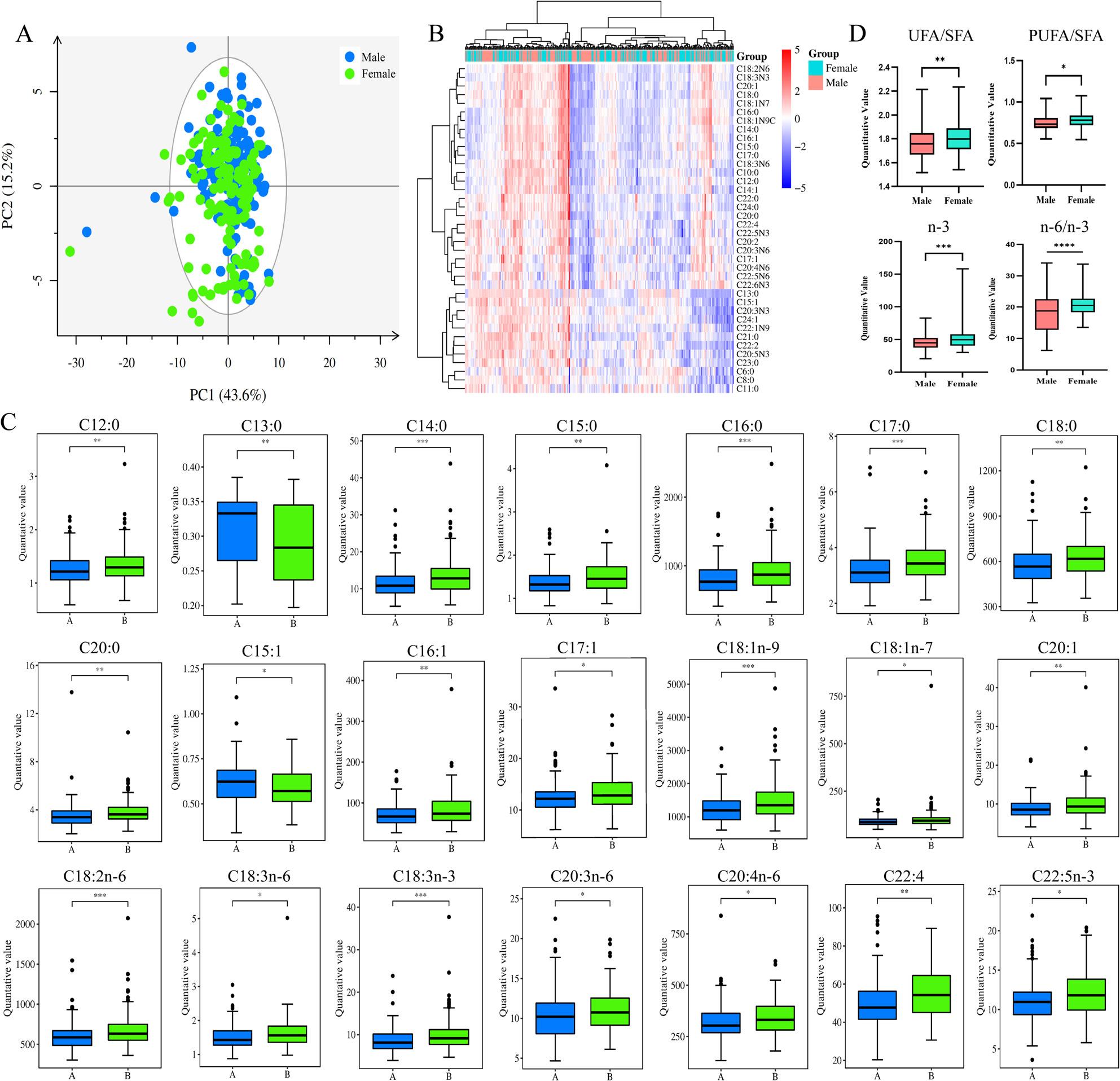
Analysis of fatty acid content differences between males and females. **A** PCA analysis. **B** Cluster analysis. **C** Box plots showed the differences in fatty acid content between males and females. On the x-axis, blue represents male, green represents female, and the y-axis represents the fatty acid content. **D** Box plots showed the differences in fatty acid statistical indicators between males and females. Significance levels are indicated as follows: * P < 0.05, ** P <0.01, *** P < 0.001, **** P < 0.0001

We performed principal component analysis (PCA) on the content of 38 fatty acids in all individuals. The results showed that fatty acid content did not show significant population stratification (Fig. 2A). In addition, cluster analysis of these fatty acid levels revealed that some individuals exhibited overall higher fatty acid content (Fig. 2B). Among the fatty acid composition traits, 25 showed significant differences between male and female ducks (*P* < 0.05) (Fig. 2C and D). Except for C13:0 and C15:1, the average contents of all other traits were significantly higher in females than in males (*P* < 0.05). Among them, the differences in C14:0, C16:0, C17:0, C18:1n-9, C18:2n-6, and C18:3n-3 between male and female ducks were highly significant (*P* < 0.001). Notably, the content of n-6/n-3 fatty acid was significantly higher in females than in males (*P* < 0.001).

### Candidate genes identified by GWAS for fatty acid composition traits

**Fig. 3:**
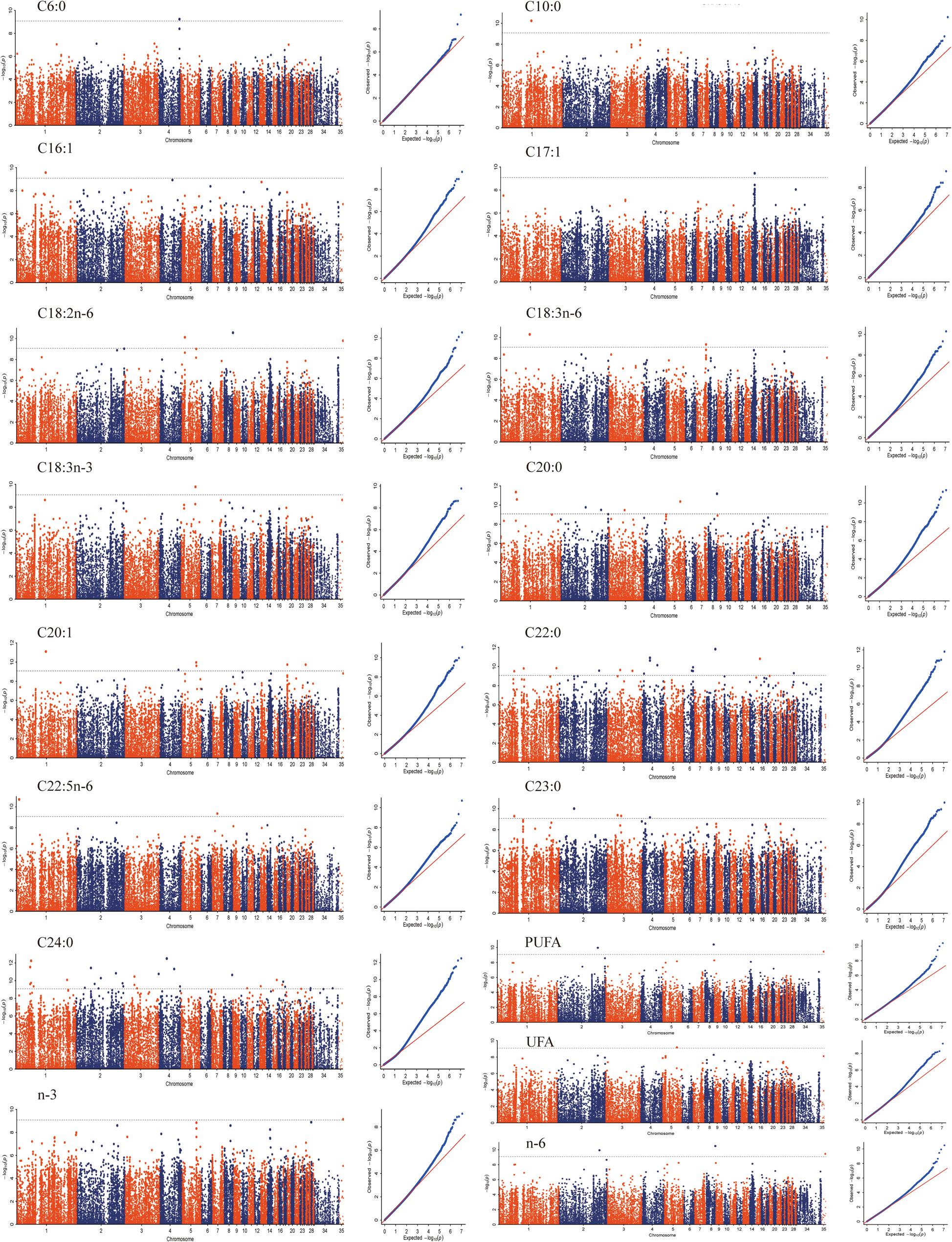
Genome-wide association analysis between genotypes and phenotypes of fatty acid traits. Manhattan (left) and QQ plots (right). The gray line represents the Bonferroni corrected significance threshold (-Log10 P = 8.38). The x-axis shows the physical positions of each marker along the chromosomes, and the y-axis shows the -Log10 P values for the association tests

The results of the GWAS analysis using fatty acid composition traits as phenotypes are shown in Supplementary Table S3 and Fig. 3. A total of 158 significant SNPs were identified (-Log_10_ *P* > 8.38), which were associated with 17 fatty acid composition traits, including C6:0, C10:0, C16:1, C17:1, C18:2n-6, C18:3n-6, C18:3n-3,C20:0, C20:1, C22:0, C22:5n-6, C23:0, C24:0, PUFA, UFA, n-3 and n-6. Among these, the SNP chr8: 32,076,308 was associated with seven different traits, while chr1: 100,428,731, chr2: 138,999,059 and chr5: 50,289,333 were each associated with five traits. In addition, 22 identical SNP loci were identified in both the C22:0 and C24:0 traits. Based on SNP annotation, a total of 70 candidate genes were identified, such as *LDLRAD3*, *PROK2*, *ACVR1*, and *FGF6*, among others.

### Functional enrichment and expression analysis of candidate genes

**Fig. 4:**
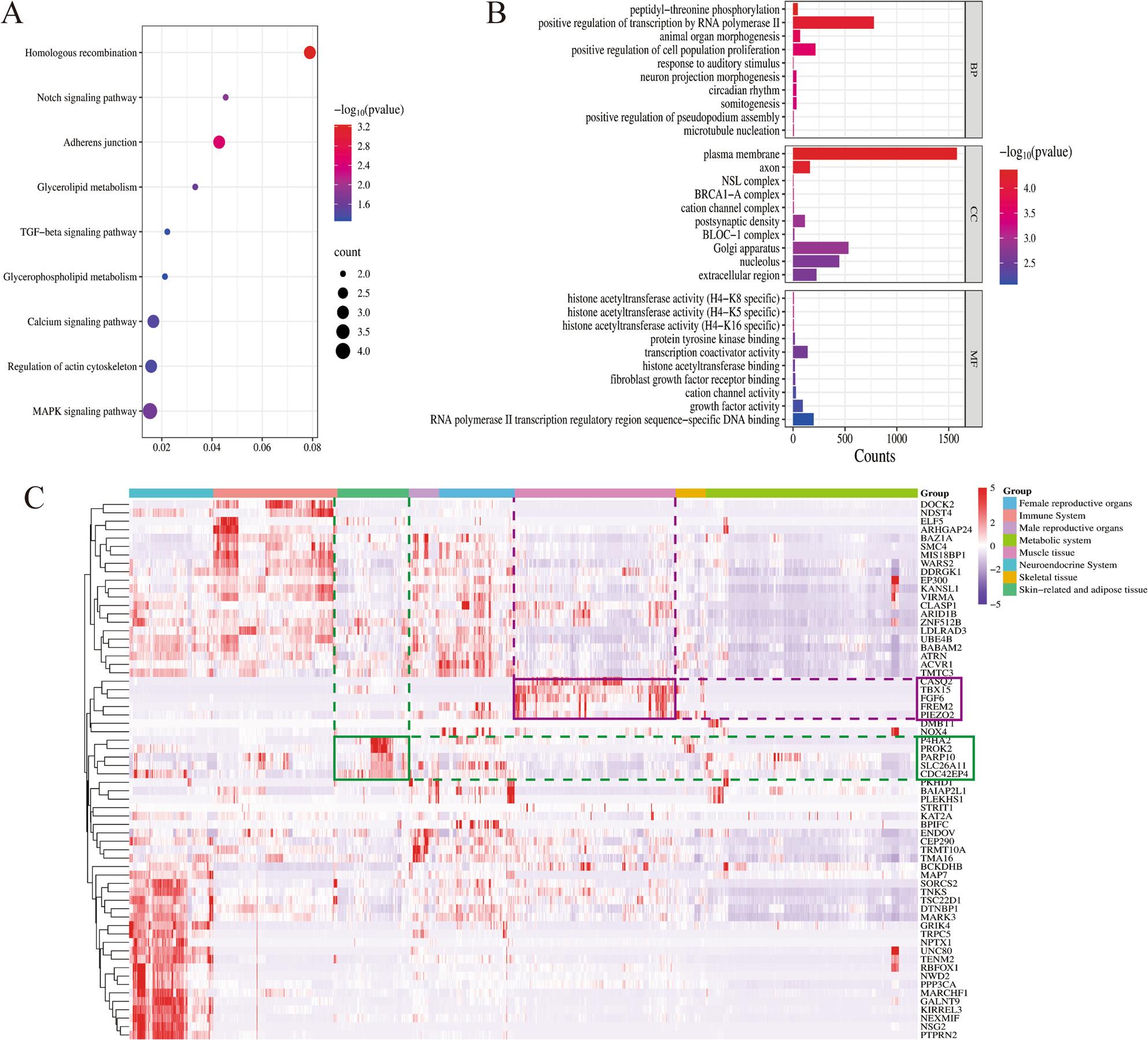
Pathway enrichment of candidate genes. **A** KEGG analysis. The x- and y-axis showed the enrich factor and pathway names. The size of the points represents the gene number. **B** GO analysis. The y-axis corresponds to the GO terms. The color of the bar represents the P value. **C** Expression heatmap of candidate genes across different duck tissues

To further explore the functional and regulatory roles of the significant SNP markers and their associated candidate genes, we performed functional enrichment analysis using the GO and KEGG databases on a total of 70 genes. The KEGG and GO analyses identified 6 significantly enriched pathways and 252 enriched GO terms, respectively (Supplementary Tables S4 and S5). Notably, pathways such as Adherens junction, Notch signaling, and Calcium signaling were significantly enriched (Fig. 4A). In the GO analysis (Fig. 4B), terms such as *positive regulation of transcription by RNA polymerase II* and *protein tyrosine kinase binding* were prominently enriched. Additionally, within the cellular component category, the *plasma membrane* and *cation channel complex* were significantly overrepresented. Then, we analyzed the expression levels of candidate genes in multiple organs and tissues including the Neuroendocrine System, Immune System, Skin-related and Adipose Tissue, Male Reproductive Organs, Female Reproductive Organs, Muscle Tissue, Skeletal Tissue, and Metabolic System. The results showed that genes *FGF6*, *TBX15*, *CASQ2*, *FREM2*, and *PIEZO2* were highly expressed in muscle tissue, while genes *P4HA2*, *PROK2*, *PARP10*, *SLC26A11*, and *CDC42EP4* were highly expressed in skin-related and adipose tissue. These genes may play important roles in regulating fatty acid metabolism in pectoral muscle (Fig. 4C).

### LD analysis of significant candidate regions for C17:1 content

**Fig. 5:**
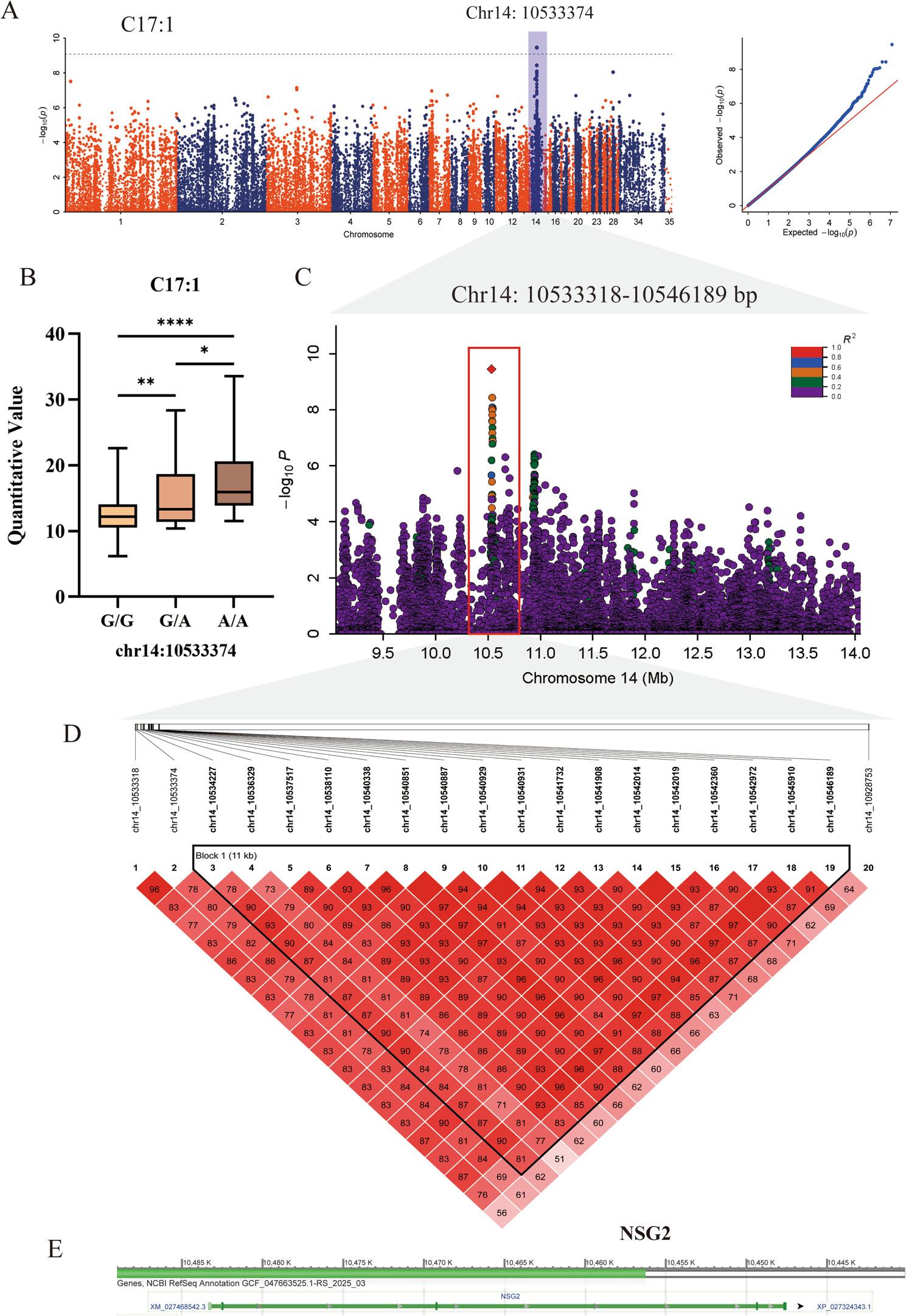
LD analysis in the region (Chr14: 10,533,318-10,546,189 bp) for C17:1 content. **A** The Manhattan plot of C17:1 content. **B** Analysis of differences in C17:1 content among lead SNP (chr14:10,533,374, G>A) genotypes. Statistical significance was defined as * P < 0.05, ** P <0.01, and **** P < 0.0001. **C** Locuszoom in the candidate region (10,533,318-10,546,189 bp). **D** LD analysis in the region (R2>0.4). **E** The genomic location of the NSG2 gene on chromosome 14

According to the GWAS results, a significant candidate region associated with C17:1 was identified on chromosome 14 (Fig. 5A). Analysis of the lead SNP (chr14:10,533,374, G > A) revealed that the AA genotype was associated with significantly higher C17:1 content compared to both GA and GG genotypes (*P* < 0.05), with the GA genotype also showing significantly higher levels than GG (*P* < 0.05) (Fig. 5B). Furthermore, this SNP accounted for 10.2% of the phenotypic variance in C17:1 content. Then, we delineated a candidate interval (10,533,318 − 10,546,189 bp) based on pairwise LD analysis around the lead SNP, within which 19 SNPs showed strong linkage with the lead SNP (R² > 0.4; Supplementary Table S6; Fig. 5C). These SNPs formed a distinct LD block (Fig. 5D), suggesting a potentially co-inherited genomic region. Finally, we screened the *NSG2* gene (Fig. 5E).

## Discussion

The content and composition of fatty acids not only influence meat quality but also play a crucial role in its nutritional profile [13]. This study detected the fatty acid composition in the breast muscle of Sansui ducks, in which the UFA (64.01%) were predominant and the major fatty acids were the C18:1n-9 (31.84%), C18:2n-6 (15.24%), and C20:4n-6 (8.16%). Meanwhile, C16:0 (20.59%) and C18:0 (14.69%) were the major SFA. These findings are consistent with previous reports on Pekin, Nonghua, and wild ducks [4, 14–16]. Notably, oleic acid (C18:1n-9) was the most abundant fatty acid in the breast muscle of Sansui, Pekin, and Nonghua ducks, whereas wild ducks had lower oleic acid and the highest C20:4n-6 content. This difference may result from the combined effects of long-term domestication, dietary regulation, and muscle adaptation. Oleic acid’s strong antioxidant properties help preserve freshness and improve flavor [17], which may contribute to the tenderness of Sansui duck meat. Additionally, Sansui ducks had higher MUFA (36.61%) than PUFA (27.40%) content, opposite to the pattern in Pekin and Nonghua ducks. High MUFA may reflect superior meat tenderness and flavor, while high PUFA, especially n-6 and n-3 fatty acids, may indicate greater nutritional value [18–20]. A lower n-6/n-3 ratio is considered more beneficial for health, with < 4:1 being optimal [21]. In this study, the ratio (21.13) far exceeded the ideal, though it was lower than in Pekin ducks and higher than in Nonghua ducks. This difference may be due to genetic background, dietary fatty acid composition, and feeding methods [22].

Morever, we compared the fatty acid profiles between male and female ducks and found that 21 fatty acids differed significantly between sexes (*P* < 0.05). Most fatty acids, as well as the PUFA/SFA and n-6/n-3 ratios, were significantly higher in females than in males (*P* < 0.05). This difference may reflect sex-specific strategies in energy allocation, lipid metabolism, and reproductive adaptation [23, 24]. Studies have reported that in Pekin ducks [25], females have higher MUFA content and n-6/n-3 ratios, while males exhibit higher PUFA/SFA ratios and n-6 levels. Similarly, in geese, n-6, PUFA, and the n-6/n-3 ratio are significantly higher in females than in males [26]. While sex is generally recognized as an important factor influencing fatty acid composition in poultry, the specific effects and outcomes vary, which is normal phenomenon due to differences in breed, nutrition, and other factors.

It can be concluded that genetic factors are important regulators of fatty acid metabolism in poultry breast muscle. Through the GWAS, genetic variation sites related to fatty acid content traits were identified, and functional and analyses suggest that *FGF6*, *CASQ2*, *FREM2*, *PIEZO2*, *PROK2*, *CDC42EP4*, *NSG2*, and *LDLRAD3* are potentially involved in muscle and lipid metabolism. During lipid metabolism, fatty acid synthesis and storage shape the overall fatty acid profile. *LDLRAD3* has been reported to regulate *de novo* lipogenesis via Sp1, SREBP, and Ap4 [27], while *ACVR1* is expressed in sheep tail and visceral fat and is involved in lipid storage [28]. Meanwhile, the proliferation and differentiation of intramuscular preadipocytes ultimately influence intramuscular fat content and fatty acid composition. Fibroblast growth factor 6 (*FGF6*) promotes the proliferation of preadipocytes, thereby maintaining adipose tissue homeostasis [29], and can also regulate energy metabolism by influencing adipocyte gene expression [30]. In mice, *PKR1* directly inhibits preadipocyte proliferation and differentiation, while prokineticin-2 (*PROK2*) can activate the PKR1 receptor [31]. *CDC42EP4*, an effector of *CDC42*, is involved in metabolic pathways including insulin, leptin signaling and adipocyte differentiation [32].

Muscle growth and oxidative capacity also influence fatty acid synthesis and conversion in the breast muscle. Calcium signaling is essential for muscle development, and *CASQ2* plays a key role in regulating Ca²⁺ release and buffering in the sarcoplasmic reticulum [33], with studies confirming its involvement in muscle growth regulation [34]. *FREM2* encodes an extracellular matrix protein and has been reported as a candidate gene affecting beef quality, with SNPs within the gene associated with carcass traits [35, 36]. These candidate genes may therefore be indirectly involved in intramuscular fatty acid metabolism, although their interactions and regulatory mechanisms remain to be elucidated. Notably, *NSG2* is a neuron-specific gene [37]. Although no studies have linked it to lipid metabolism, the most significant SNP associated with C17:1 (chr14:10,533,374) explains 10.2% of the phenotypic variation, suggesting it may influence C17:1 metabolism either by regulating nearby genes or through NSG2 affecting other lipid metabolism genes.

The ZJU1.0 version used in this study is a valid and high-quality duck reference genome, although a newer T2T assembly of the duck reference genome has since been released. Its sequence and all associated annotation information are still accessible and available for download from the NCBI database, and it continues to be widely used in recent studies [38–42]. Moreover, using ZJU1.0 ensured continuity with our previous research [43], facilitating the integration of genetic findings across multiple independent experiments and thereby strengthening the cumulative evidence base of this study. The complete analytical pipeline has been made publicly available to enable other researchers to validate and expand upon our findings using updated genome assemblies.

Fatty acids play a crucial role in meat quality and flavor. In this study, GWAS identified several SNPs and candidate genes associated with fatty acid content in duck breast muscle. Although fatty acid regulation and metabolism are complex and the direct evidence obtained so far remains limited, these SNPs and candidate genes provide valuable theoretical references for molecular breeding aimed at improving duck meat quality. Future studies integrating functional validation with genomic selection may elucidate the roles of these SNPs and candidate genes in fatty acid deposition and metabolism, thereby providing a solid foundation for the molecular breeding of high-quality Sansui duck meat and offering novel insights into lipid metabolism in poultry.

## Conclusions

This study investigated the fatty acid composition of Sansui duck breast muscle and identified C18:1n-9, C16:0, C18:2n-6, C18:0, and C20:4n-6 as the predominant fatty acids. Female ducks exhibited significantly higher levels of several key fatty acids compared to males. GWAS revealed 158 SNPs significantly associated with fatty acid composition traits, annotated to 70 protein-coding genes, including *LDLRAD3*, *ACVR1*, *FGF6*, and *CASQ2*. Furthermore, LD analysis identified a candidate region (10,533,318–10,546,189 bp) on chromosome 14 and a key candidate gene (*NSG2*) which may influence C17:1 content. Enrichment analysis highlighted pathways such as Adherens junctions and MAPK signaling in fatty acid biosynthesis. These results provide insights into the genetic basis of lipid metabolism and meat quality improvement in ducks.

## Materials and methods

### Animals and sampling

A total of 350 ducks (180 males and 170 females) were provided by the Sansui Duck Breeding Farm of the Institute of Animal Husbandry and Veterinary Medicine, Guizhou Academy of Agricultural Sciences. The feeding and management process of all Sansui ducks were the same. The whole process is mixed breeding of male and female, and at the same time, immunization prophylaxis is carried out according to the regular immunization program of ducks. At 20 weeks of age, 4 mL of blood samples were collected from 305 experimental ducks (155 males, 150 females and 45 dead or number missing) and stored at -20℃. All ducks were sacrificed at 20 weeks of age by exsanguination. Breast muscles were collected and stored in a 4℃ refrigerator.

The animal study protocol was reviewed and approved by the animal care and welfare committee of the Institute of Animal Husbandry and Veterinary Medicine, Guizhou Academy of Agricultural Sciences (Grant No.202304/2023-07) and performed in accordance with National Institute of Animal Health guidelines for animal experiments.

### Determination of fatty acid content

Duck breast muscle tissue samples were homogenized in a chloroform-methanol (2:1) solution using a high-throughput tissue grinder with glass beads. After centrifugation, the supernatant was collected and subjected to esterification with 1% sulfuric acid-methanol at 80 °C for 30 min. The resulting fatty acid methyl esters were extracted with n-hexane, washed with cold water, and dehydrated with anhydrous sodium sulfate. Methyl salicylate (500 ppm) was added as an internal standard prior to GC-MS analysis.

Gas chromatographic analysis was performed using a Trace 1300 gas chromatograph (Thermo Fisher Scientific, USA) equipped with a Thermo TG-FAME capillary column (50 m × 0.25 mm ID × 0.20 μm). Helium served as the carrier gas at a constant flow rate of 0.63 mL/min. Sample injection was conducted in split mode (8:1) with an injection volume of 1 µL and an injector temperature of 250 °C. The temperatures of the ion source and transfer line were maintained at 300 °C and 280 °C, respectively. The column temperature was programmed as follows: initial temperature of 80 °C held for 1 min, increased to 160 °C at 20 °C/min and held for 1.5 min, then raised to 196 °C at 3 °C/min and maintained for 8.5 min, and finally increased to 250 °C at 20 °C/min and held for 3 min. Mass spectrometric detection was carried out on an ISQ 7000 system (Thermo Fisher Scientific, USA) in electron impact ionization mode with an electron energy of 70 eV, operating in selected ion monitoring (SIM) mode [44, 45].

### Phenotyping

The fatty acid content of 38 different types was measured in the breast muscle of 297 Sansui ducks using gas chromatography (Remaining 8 individuals had loss of pectoral muscles or undetectable pectoral fatty acids). Additionally, 12 fatty acid statistical indicators were calculated. In total, 50 fatty acid composition traits were obtained, as detailed in Supplementary Table S1 and S2. Before the association analysis, fatty acid composition traits exhibiting non-normal distributions were normalized using a log₂ transformation. The resulting normalized data were thereby employed in all subsequent association analyses to enhance the reliability and robustness of the statistical inferences.

### Genome re-sequencing and SNP genotyping

DNA was extracted from the duck’s blood (*n* = 305) using the phenol-chloroform protocol. DNA quality was detected by Nano Drop-2000 and agarose gel electrophoresis. After being examined, standard procedures generated paired-end libraries for each eligible sample. In addition, the average insert size was 450 bp. All libraries were sequenced on a DNBSEQ-T7 platform (BGI, China) using 150 bp paired-end reads, achieving an average sequencing depth of 10×. The original raw data file still contains interference information, such as connectors and low-quality reads. To meet the needs of subsequent analysis, this study uses Trimmomatic to filter raw data to obtain clean reads [46]. The filtering conditions are (1) filtering out the joint sequence. (2) Delete reads with unknown base pair N content greater than 10%. (3) Delete reads less than 25 bp in length. (4) Remove low-quality (base mass value less than Q20) reads.

The clean reads obtained were then compared with the duck reference genome (ZJU1.0) using Burrows-Wheeler Aligner (BWA aln) [47]. HaplotypeCaller in GATK was used to identify and analyze SNPs and VCFtools was used to filter SNP data further [48, 49]. Finally, a total of 11,947,837 SNPs were obtained by VCFtools for GWAS analysis. SNPs were filtered based on the following criteria: (1) SNPs had to have a minor allele frequency > 0.05 and a major allele frequency < 0.99; (2) the maximum missing rate was < 0.1; and (3) SNPs could only have two alleles.

### Genome-wide association analysis (GWAS)

Genome-wide association studies (GWAS) were conducted using the mixed-effects linear model program Emmax to identify SNPs [50]. The model was formulated as follows:

$$\:y=X\alpha\:+Z\beta\:+W\mu\:+e$$

In this formula, y represents the vector of phenotypic observations; Xα represents a fixed effect vector for modified population stratification, including gender the first three principal component values (PCA feature vectors) of genome-wide SNP genotypes, to correct population stratification [51]; Zβ represents the effect of SNPs; W represents random animal effects, and the variance covariance matrix is based on a kinship matrix based on genotype-based whole-genome SNP genotype estimation; e indicates the random residual vector. Manhattan and QQ plots were generated using the R package (version 4.5.1). The genome-wide significance threshold was established through Bonferroni correction, a stringent method chosen to control the family-wise error rate in the context of massive parallel testing. The threshold was set at *P* < 4.18 × 10^− 9^ (corresponding to -log_10_*P* = 8.38), calculated as 0.05 divided by the total number of independent SNPs (119,478,37). Additionally, QQ plots were used to evaluate the potential inflation of false positives due to population stratification.

### LD analysis

VCFtools was employed to extract individual genotypes within regions of interest. Linkage disequilibrium (LD) analysis among the most significant SNPs in the significant candidate region was performed using PLINK (version 1.90). The Locuszoom plots were generated using R (version 4.5.1). Additionally, Haploview [52] software was utilized to assess the overall LD pattern.

### Gene enrichment and expression analysis

Based on the duck reference genome (ZJU1.0), significant SNPs were functionally annotated using SnpEff software [53] to identify candidate genes associated with various traits. Gene Ontology (GO) and Kyoto Encyclopedia of Genes and Genomes (KEGG) pathway enrichment was conducted using KOBAS 3.0 (http://bioinfo.org/kobas) [54]. A threshold of *P* < 0.05 was applied to determine statistically significant enrichment for both GO terms and KEGG pathways. We performed expression level analysis of the screened candidate genes by downloading published gene expression matrices from various duck tissues [55].

### Statistical analysis

Microsoft Excel 2021 was used to calculate the mean and standard deviation for each trait. Statistical significance of trait differences between males and females was assessed using SPSS software (version 22.0). Correlation analysis, PCA, and cluster analysis among various traits were conducted using RStudio (version 4.5.1), and heatmaps were visualized. Graphs were generated with GraphPad Prism (version 8.0.2) and RStudio.

## Supplementary Information

Supplementary Material 1

## Data Availability

The raw genome sequencing data have been deposited in the Genome Sequence Archive (Genomics, Proteomics & Bioinformatics 2025) in National Genomics Data Center (Nucleic Acids Res 2025), China National Center for Bioinformation / Beijing Institute of Genomics, Chinese Academy of Sciences (GSA: CRA036051) at https://ngdc.cncb.ac.cn/gsa/browse/CRA036051.
